# Emerging infectious disease outbreaks in Sub-Saharan Africa: Learning from the past and present to be better prepared for future outbreaks

**DOI:** 10.3389/fpubh.2023.1049986

**Published:** 2023-05-09

**Authors:** Enos Moyo, Malizgani Mhango, Perseverance Moyo, Tafadzwa Dzinamarira, Itai Chitungo, Grant Murewanhema

**Affiliations:** ^1^School of Nursing and Public Health, University of KwaZulu-Natal, Durban, South Africa; ^2^School of Public Health, University of Western Cape, Bellville, South Africa; ^3^Faculty of Medicine and Health Sciences, Stellenbosch University, Stellenbosch, South Africa; ^4^School of Health Systems and Public Health, University of Pretoria, Pretoria, South Africa; ^5^College of Medicine and Health Sciences, University of Zimbabwe, Harare, Zimbabwe

**Keywords:** emerging infectious diseases, Sub-Saharan Africa, public health response, preparedness and response, outbreak

## Introduction

Over the past two decades, Sub-Saharan Africa (SSA) has faced multiple public health emergencies (1). A public health emergency (PHE) is a situation with health consequences too severe for conventional community response (2). Between 2001 and 2022, the region reported 1,800 PHEs, most of them emerging infectious diseases (3). Emerging infectious diseases are new or resurgent diseases in the population (4). Cholera, meningitis, Ebola, measles, yellow fever, monkeypox, Zika, Rift valley fever, and COVID-19 were some of the reported emerging infectious diseases (5). Multiple factors contribute to the rise in SSA's emerging infectious diseases. These include microorganisms adapting to climate and weather changes, shifting ecosystems, and human susceptibility to infection due to immunosuppression, malnutrition, and poor immunization (4).

The region has also seen a rise in zoonotic pathogen outbreaks caused by monkeypox and Ebola viruses. From 2012 to 2022, zoonotic disease outbreaks in SSA rose 63% compared to 2001–2011. From 2001 to 2022, 33% of PHEs were zoonotic disease outbreaks (3). Almost 70% were caused by Ebola virus disease (EVD) and other viral hemorrhagic fevers and 30% by dengue fever, anthrax, plague, monkeypox, and others (3). A growing population driving greater demand for animal-derived food is associated with zoonotic disease outbreaks. Population growth and urbanization have reduced wildlife habitats. SSA has the lowest per-capita health spending and healthcare worker (HCW) availability, which exacerbates health problems and makes emerging infectious disease outbreaks difficult to manage (4). In this opinion piece, we discuss the problems SSA encountered during prior and current emerging infectious disease outbreaks, the strengths realized, and our recommendations to better prepare the region for future emerging infectious disease outbreaks.

## Challenges faced during previous emerging infectious disease outbreaks

In this section, we unpack challenges faced during previous outbreaks grouped into individual, health system, and community-level challenges.

### Individual-level challenges

Poor socioeconomic status (SES), stigma, low-risk perception, limited access to healthcare, and cultural beliefs contribute to emerging infectious disease outbreaks in SSA (6). About 34% of SSA households were in extreme poverty living on <$1.90/day in 2021 (7). Overcrowding and the resultant poor hygiene have been associated with emerging infectious diseases such as cholera, measles, monkeypox, EVD, and COVID-19 (8, 9). About 20% of the African population was undernourished in 2019 (10), thereby increasing population vulnerability to infectious diseases, and reducing treatment response due to diminished immunity (11, 12).

Poor SES is linked to low health literacy, limiting infection prevention and control knowledge. Reduced awareness leads to fewer immunizations and delayed treatment, possibly contributing to emerging infectious disease spread (13). Some people have a low-risk perception and don't take infectious disease precautions (8). Delays in seeking medical care raise the community disease burden (14, 15). Some traditional burial practices such as washing dead bodies contributed to the rapid spread of EVD in West Africa during the 2014–2016 outbreak (6).

### Health system level challenges

Underinvestment in health (16), conflicts (5, 17, 18), inadequate disease surveillance systems (6, 19), and a surge in non-communicable diseases (18) disrupted SSA healthcare systems. Critical shortages of HCWs, personal protective equipment (PPE), and testing platforms created weaknesses in active surveillance. The lack of electronic databases contributed to poor data quality due to inconsistency and incompleteness (5, 18). Several SSA countries are categorized as fragile states, and they are struggling to grow due to weak institutions, bad governance, political instability, and frequently ongoing violence or the after-effects of previous conflicts (17). Conflicts disrupt healthcare delivery due to the closure of healthcare facilities, loss of medical equipment, and migration of HCWs (17).

Poor disease surveillance has been attributed to a lack of experienced HCWs, heavy workloads, and low HCW motivation. The lack of PPE and isolation rooms complicates infection control, resulting in patient-to-HCW cross-infections (6). Low laboratory testing capacity in SSA's vulnerable populations contributes to virus spread (20). PHEs disrupt normal childhood immunizations, resulting in disease outbreaks (21).

### Community-level challenges

People unlawfully cross SSA's porous borders (22). Without effective port-of-entry surveillance, persons can import/export emerging infectious diseases across countries, complicating contact tracing (6). Mistrust of governments and the healthcare system led to a drop in immunization and COVID-19 treatment (23). Furthermore, even some people may not seek healthcare for fear of social stigma as was common in the EVD-affected communities in Sierra Leone, Guinea, and Liberia (6).

## Strengths associated with the management and control of previous emerging infectious disease outbreaks

There were also some notable strengths, which we stratified into individual-level, health system-level, and community-level strengths.

### Individual-level strengths

In 2018, SSA had 44% mobile phone penetration (24). Cell phone technology has made social media platforms easier to use. This means that more people can now be reached with messaging about interrupting infection chains and refuting rumors, misunderstandings, and misconceptions about emerging infectious diseases (25). The relatively youthful populations in SSA meant that those who got the infections recovered quickly if there were no underlying immunosuppressive conditions (1).

### Health system level strengths

Several SSA nations have had an emerging infectious disease outbreak, therefore they have response strategies for PHEs. HCWs with prior experience in handling emerging infectious disease outbreaks are valuable in managing ongoing emerging infectious disease outbreaks (26). As part of emergency response operations in SSA, non-governmental organizations (NGOs) provided technical, financial, and logistical aid to health facilities, reducing the financial load on afflicted countries (27).

During the EVD and COVID-19 epidemics, SSA nations cooperated to improve disease testing (28). The majority of SSA nations also have community health workers who were employed during prior emerging infectious disease outbreaks to warn their communities and health authorities about probable cases (29). Instead of focusing on contacts, mass immunization initiatives showed the potential in limiting emerging infectious diseases (30, 31).

During COVID-19, SSA increased genomic surveillance, and South Africa and Botswana detected the Beta and Omicron variants. Transparency in reporting revealed SSA countries were ready to collaborate to control emerging infectious diseases (32). Despite numerous challenges, there has been progress in the utilization of the integrated disease surveillance and response (IDSR) framework in SSA. In the past two decades, 94% of the countries used the framework to enhance surveillance capacity for priority diseases (19).

### Community-level strengths

Past PHEs in Africa resulted in structures for future emerging infectious disease outbreaks (6). Re-engaging local community members who had been trained to distribute information about the EVD outbreak in the Republic of Congo boosted community awareness of the messaging. Communities accepted HCWs, tolerated prescribed behaviors, and felt more comfortable reporting cases (30). During the EVD outbreak in Cote d'Ivoire, community-led education about infection control helped people wash their hands more often and stop unhealthy practices associated with shaking hands and burying their dead. This education was then used during subsequent outbreaks (30).

## Recommendations on how SSA can be better prepared for future emerging infectious disease outbreaks

From the challenges and strengths experienced during the previous and current emerging infectious disease outbreaks, we came up with recommendations on how SSA might better prepare for future emerging infectious disease outbreaks.

### Individual-level recommendations

SSA's low SES worsens conflict and disease outbreaks. SSA countries should come up with plans to raise their populations' SES. Strategies include increasing industrialization, maximizing agricultural potential, and integrating regional markets (33). Improved economic activity creates jobs, improves food security, and increases investment in basic utilities like water and sanitation, reducing emerging infectious disease outbreaks (7). Table 1 summarizes each strategy and the required actions.

**Table 1:** Strategies to improve the population's socio-economic status and strengthen health systems.

**Strategies to improve the population's health**	
**Strategy**	**Actions required**
Increasing industrialization	• Attracting investment through good economic policies • Governments should increase infrastructure development • Partnering with the private sector to pool resources to fund industrialization • Local manufacturing • Value addition to raw materials before export
Maximizing agricultural production	• Increase farmer's access to credit • Mechanization of the sector • Improve the land tenure system
Integration of regional markets	• Building regional infrastructure • Facilitate easy movement and employment of people across borders • Encouraging intra-African trade and investment
**Strategies to strengthen health systems**	
**Health system pillar**	**Strengthening strategies**
Financing	• Public-private partnerships • Taxation
Service delivery	• Implementation of universal health coverage • Decentralization of services
Workforce	• Intensify HCW training and retention strategies • Avail opportunities for HCW professional development • Opportunities for HCWs in remote areas to be able to consult experts when they encounter difficult cases
Health Information Systems	• Training of HCWs in the use of ICT • Integration of local and national health management information systems
Access to essential medicines	• Rational use of essential medicines • Stewardship of antibiotics • Evidence-based selection, procurement, and supply management of essential medicines and devices
Leadership	• HCWs should have leadership training • Implementation of participatory and distributed leadership

We advocate improving access to education and initiating health education in preschool to increase health literacy. With more health knowledge, the population's risk perception may rise, leading to more vaccinations and healthcare utilization (34).

### Health system level recommendations

The health system recommendations include the implementation and strengthening of the One Health approach, strengthening health systems, and improving disease surveillance.

#### One Health approach

SSA countries should strengthen the One Health Approach. One Health Approach addresses zoonoses where people, animals, and the environment intersect. It aims to create interdisciplinary, cross-sectoral, and cross-regional collaboration to predict emerging infectious disease outbreaks (35). Domestic and wild animals harbor diseases that cause emerging infectious diseases. Integrated human, animal, and environmental surveillance can explain pathogen-sharing paths, allowing for more complete treatments that prioritize prevention at the source. Increasing cross-sectoral cooperation can help encourage science-based decision-making, reduce wasteful duplication, and address external variables driving disease burden (36). Animal handlers are prone to emerging infectious diseases and often disseminate new diseases to other people. Regular surveillance of these individuals is critical in preventing emerging infectious disease outbreaks. To improve early warning systems and emerging infectious disease outbreak detection, proactive animal handler surveillance can be an important addition to hospital surveillance (37).

#### Strengthening health systems

Sub-Saharan Africa should enhance its health systems to handle future emerging infectious disease outbreaks. Workforce, service delivery, health information systems, access to essential medicines, health financing, and leadership and governance should be reinforced. These initiatives should be localized (3, 5, 38–41). Table 1 shows the strategies that can be used to strengthen the health systems in SSA.

#### Strengthening disease surveillance

Laboratories' capacity needs to be improved to be able to identify a variety of pathogens. Furthermore, it is important to create laboratory networks that link national, regional, and research reference laboratories (38). More HCWs should receive training in disease surveillance and epidemiology to improve emerging infectious disease surveillance in SSA. Refresher courses or the development of online public health programs that are flexible for full-time HCWs could accomplish this. A robust public health workforce should be formed to collect, access, share, and act on high-quality data, including making use of cutting-edge technologies like genetic sequencing and informatics.

SSA countries must upgrade their information, communication, and technology (ICT) infrastructure to gather and send patient health data. By reducing data volume and automating data collection, validation, and analysis, ICT can reduce errors. Local health management information systems (HMIS) should be integrated with national HMIS, and data should be used at every level of the healthcare system to enable ICT uptake and use. Data should be aggregated and summarized to offer summary indicators for planning and resource allocation (19). The national HMIS should have multiple sources. Antimicrobial resistance surveys, systematic surveys, environmental data, vital statistics, civil registrations, vital statistics, and research data are examples of these sources (19).

Big data and artificial intelligence (AI) can transform emerging infectious disease surveillance and response by improving the current traditional systems and strengthening outbreak detection and response (11). Digital disease surveillance (DDS) data from outside the public health system should be used. DDS aggregates and analyzes internet data unrelated to patient conditions or medical contacts. This information is available on search engines, social media, and mobile devices. DDS improves the timeliness and breadth of monitoring information in high-income countries. Thailand and New Zealand used DDS to detect, trace, and isolate COVID-19 cases (19). Community-based surveillance (CBS) should be promoted because it leads to early detection and rapid reporting of emerging infectious disease cases to the health system. Community-based surveillance helps detect risk groups and enumerate cases (42).

### Community-level recommendations

SSA countries should inform local populations about emerging infectious diseases using billboards, mainstream media, and social media platforms in their native languages (25). Social scientists should develop a strong capacity for community engagement to change unhealthy behaviors such as eating uncooked or raw food, as well as promiscuous sexual behavior (38). Engaging communities improve priority planning, resource allocation, and adoption of emerging infectious disease-related healthcare treatments, such as immunizations. It also deepens discussions and raises accountability.

## Conclusion

SSA has had multiple PHEs in the past two decades, most of them emerging infectious disease outbreaks. Emerging infectious disease outbreaks were caused by microorganism adaptation and alteration due to climatic and environmental changes, shifting habitats, and increased human susceptibility to infection due to immunosuppression, undernourishment, and poor vaccination. The bulk of emerging infectious disease outbreaks was caused by zoonotic pathogens, and their rise has been related to urbanization, a fast-expanding population, increased demand for animal food, and invasion of natural habitats. When managing and containing the emerging infectious disease outbreaks, the countries in the region encountered several challenges at the individual, health system, and community levels. Furthermore, the management and control of these outbreaks also revealed several strengths the region has. Based on these challenges and strengths, we suggest that the region implement and strengthen the One Health approach, improve the socioeconomic status of the population, strengthen its health systems, improve disease surveillance, and involve and educate communities about emerging infectious diseases.

## Author contributions

EM and MM: conceptualization and writing original draft. PM: writing original draft. TD: conceptualization, supervision, and writing review and editing. IC and GM: writing review and editing. All authors contributed to the article and approved the submitted version.
